# The relationship between copper intake and chronic kidney disease/diabetic kidney disease: insights from NHANES data (2011-2018)

**DOI:** 10.3389/fendo.2025.1674439

**Published:** 2025-10-27

**Authors:** Shasha Hu, Xiao Tian, Zhenzhen Wang, Mengmeng Li

**Affiliations:** ^1^ Department of Nephrology, Zibo Central Hospital, Zibo, China; ^2^ Department of Critical Care Medicine, Zibo Central Hospital, Zibo, China; ^3^ Department of Endocrinology, Zibo Central Hospital, Zibo, China

**Keywords:** chronic kidney disease, diabetic kidney disease, copper intake, NHANES, relationship

## Abstract

**Background:**

The connection between copper consumption and chronic kidney disease (CKD), as well as diabetic kidney disease (DKD), is still unclear. This research seeks to explore the link between copper intake and both CKD and DKD by analyzing data from the National Health and Nutrition Examination Survey (NHANES), which was carried out from 2011 to 2018.

**Methods:**

Participant data were derived from the 2011–2018 NHANES database. For evaluating variable differences, complex sampling design - weighted t - tests were used for continuous variables, and weighted chi - square tests for categorical ones. Univariate regression analysis probed the relationship between variables and the dependent variable. Multivariate logistic regression analysis was employed to establish a multivariable model. Restricted Cubic Spline (RCS) curves were applied to explore the potential nonlinear association of copper intake with CKD and DKD.

**Results:**

The study involved 16,948 participants, comprising 3,319 individuals with CKD and 13,629 controls without CKD. The findings from the multivariate logistic regression analysis demonstrated negative correlation between copper intake and CKD (OR > 0.80) and DKD (OR > 0.5). This protective effect was observed consistently across all analyzed population subgroups. Furthermore, the RCS analysis suggested a possible non-linear association between copper intake and CKD and DKD. An inverse association was observed between copper intake and CKD in the group with copper intake ≤ 1.47 mg (OR = 0.51), and between copper intake and DKD in the group with intake ≤ 0.98 mg (OR = 0.40).

**Conclusion:**

Copper intake was found to be significantly negatively associated with CKD and DKD within a certain range.

## 1 Introduction

Chronic kidney disease (CKD) is a gradually progressive and irreversible condition characterized by persistent loss of kidney function and concomitant structural damage (1, 2). It is more prevalent among older adults, women, racial and ethnic minority groups, as well as individuals with diabetes mellitus or hypertension (3). The growing number of CKD cases worldwide is partly due to the increase in risk factors like obesity and diabetes mellitus, with approximately 843.6 million people affected in 2017 (4). The primary cause of CKD is diabetic kidney disease (DKD), which arises from metabolic and hemodynamic abnormalities associated with prolonged diabetes (5, 6). CKD and diabetes impose a substantial economic burden on healthcare systems worldwide, contributing significantly to social and financial costs (7, 8). Moreover, both conditions are established critical risk factors for cardiovascular disease and play a major role in global morbidity and mortality (7, 8). Given the complexity of the pathogenesis of CKD and DKD, it is particularly important to identify factors that influence the onset of these conditions.

Growing evidence indicates that overall dietary patterns may influence the development and progression of CKD (9). The intake of many dietary components is often interrelated; for example, copper-rich foods—such as nuts, seeds, and whole grains—are frequently part of diets that are also high in fiber, antioxidants, and other beneficial micronutrients (10). Copper is an essential trace mineral for the human body, playing a critical role in various physiological processes, including erythropoiesis, immune function, and energy metabolism (11, 12). However, excessive exposure to copper has been linked to the development of several chronic diseases, such as atherosclerosis, coronary heart disease, Alzheimer’s disease, and diabetes (13–15). Previous studies have shown that increased circulating copper mirrors are associated with CKD (16, 17). Additionally, Ahmad et al. have found that high circulating copper is linked to the prevalence of CKD and a reduction in the estimated glomerular filtration rate (eGFR) (18). Recent studies have discovered that the concentration of urinary copper in patients with DKD is significantly higher than that in non-DKD patients (19). Wu et al. have demonstrated that serum copper levels are elevated in diabetic patients (20). Serum copper concentration is significantly associated with the risk of diabetes in hypertensive individuals (20). These findings indicate that copper represents a crucial risk factor for the development of diabetes. Furthermore, another study found that as copper intake increased, the risk of diabetic nephropathy decreased (21). Thus, it is important to explore the association between dietary copper intake and the prevalence of CKD and DKD.

## 2 Methods

### 2.1 Study population

**Figure 1:**
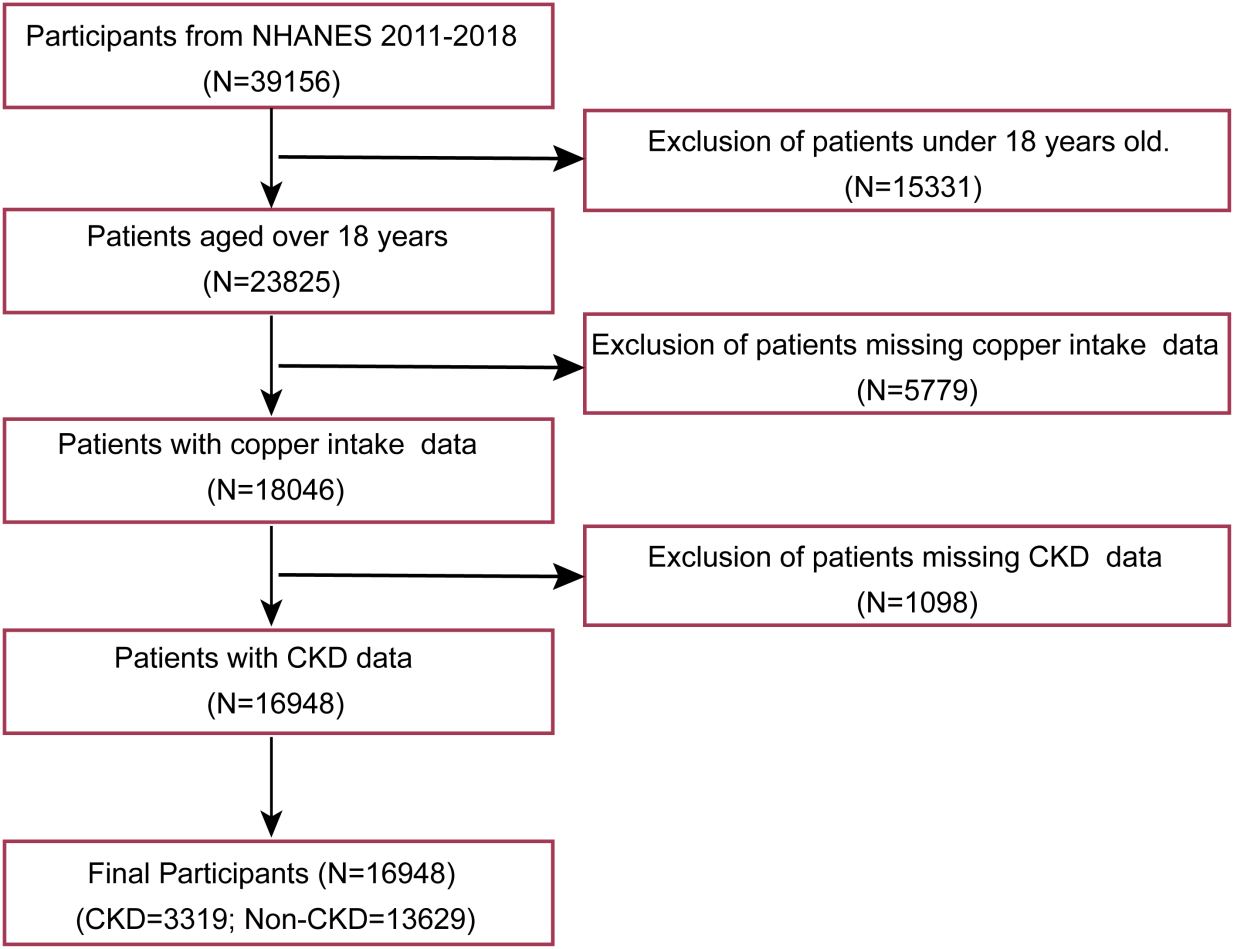
Patient selection flowchart. NHANES: national health and nutrition examination survey; CKD: Chronic kidney disease.

The analysis incorporated eight years of continuous NHANES data (2011–2018 cycles), representing the most recent comprehensive national health metrics available. Using a multi-stage probability sampling design, NHANES collects demographic, dietary, and clinical examination data from non-institutionalized U.S. residents across diverse geographic regions. From an initial pool of 39,156 survey respondents, we excluded: (1) minors (n=15,331), (2) participants without recorded copper consumption data (n=5,779), and (3) cases lacking CKD status information (n=1,098). The final analytical cohort comprised 16,948 eligible adults. The participant selection flowchart is illustrated in **Figure 1**.

### 2.2 Assessment of CKD and DKD

Individuals exhibiting a urinary albumin-to-creatinine ratio (ACR) of 30 mg/g or higher (22) or an estimated glomerular filtration rate (eGFR) below 60 mL/min/1.73 m² (23) were classified as having CKD. For the purposes of this research DKD is characterized as chronic kidney disease occurring alongside diabetes mellitus. Diabetes itself was identified through self-reported diagnoses, the usage of diabetes-related medications or insulin, hemoglobin A1c levels of 6.5% or higher, or fasting glucose readings equal to or exceeding 7.0 mmol/L (23).

### 2.3 Independent variable

Each participant in the NHANES study completed two separate 24-hour dietary recall interviews. These interviews generated two types of dietary intake data: individual food records and comprehensive nutrient intake files. For each participant each day, the study assesses the total energy and nutrient intake from food and drinks, using information from the comprehensive nutrient intake files. The average copper intake (in micrograms, mcg) across both days served as the independent variable in this analysis. Participants were categorized into four groups based on quartiles: Q1 (mcg < 0.8), Q2 (mcg 0.8–1.07), Q3 (mcg 1.07–1.43), and Q4 (mcg > 1.43).

### 2.4 Dietary intake

The Healthy Eating Index-2020 (HEI-2020) is a dietary quality assessment tool developed by the U.S. Department of Agriculture (USDA) based on the “Dietary Guidelines for Americans, 2020-2025”. It quantifies the adherence of an individual’s or population’s diet to recommended dietary patterns. In this study, the HEI-2020 score was calculated from two days of dietary recall data. The intake levels for each component were determined using a standard food composition database (e.g., USDA FoodData Central) and then scored. The total score ranges from 0 to 100, with a higher score representing greater adherence. The index includes 13 components: nine adequacy components (total vegetables, greens and beans, total fruits, whole fruits, whole grains, dairy, total protein foods, seafood and plant proteins, and fatty acids) and four moderation components (sodium, refined grains, saturated fats, and added sugars). The specific scoring criteria are detailed in **Supplementary Table S1**.

### 2.5 Covariate

Covariable in this study included age, gender, race, education level, poverty income ratio (PIR), marital status, body mass index (BMI), high blood pressure (HBP), smoking status, alcohol consumption, diabetes mellitus, hyperlipidemia, biochemical indicators, and HEI-2020.

Age was classified into two distinct groups: 18–60 years and those aged 60 years or older. Race and ethnicity were categorized into five distinct groups: non-Hispanic White, non-Hispanic Black, Mexican American, other Hispanic, and other racial categories. Levels of educational attainment were divided into three classifications: less than high school, high school diploma or its equivalent, and college graduates or those with higher education. The PIR was segmented into three levels: PIR less than 1.5, PIR ranging from 1.5 to 3.5, and PIR exceeding 3.5. Marital status was classified as: married or cohabiting, widowed, divorced, separated, or never married. Body mass index (BMI) categories were established as BMI less than 25, BMI between 25 and 30, and BMI greater than 30. Smoking status was characterized as follows: never smoked (fewer than 100 cigarettes lifetime), former smoker (100 or more lifetime cigarettes but currently not smoking), and current smoker (100 or more lifetime cigarettes with active usage). Alcohol intake was grouped into three categories: heavy drinking (three or more drinks daily for women or four or more drinks daily for men), moderate drinking (two drinks daily for women or three drinks daily for men), and light or non-drinking (less than two drinks per day for women or fewer than three drinks per day for men). HBP was indicated by systolic blood pressure (SBP) of 140 mmHg or higher, diastolic blood pressure (DBP) of 90 mmHg or higher, or the current use of medications to control hypertension. Diabetes mellitus was determined if participants were using antidiabetic medications or if they met specific laboratory criteria (fasting plasma glucose of 7.0 mmol/L or greater or glycated hemoglobin [HbA1c] above 6.4%). Hyperlipidemia diagnosis rested on grounds of lipid - lowering drug use or abnormal lipid parameters, namely, total cholesterol over 200 mg/dL, triglycerides beyond 150 mg/dL, HDL - C under 40 mg/dL in men or 50 mg/dL in women, and LDL - C surpassing 130 mg/dL. As for biochemical markers, urinary ACR, determined by dividing urinary albumin (mg/dL) by urinary creatinine (g/dL), and eGFR, gauged in mL/min/1.73 m², were taken into account. HEI-2020 score were classified into two distinct groups: a good adherence group (score ≥60) and a poor adherence group (score <60).

### 2.6 Assessment of nonlinear relationships using restricted cubic splines

To explore the potential nonlinear relationship between copper intake and CKD and DKD, RCS with four knots placed at the 5th, 35th, 65th, and 95th percentiles were incorporated into logistic regression models. The analysis used a copper intake level of 1.075 mg as the reference for calculating hazard ratios (HRs) and corresponding 95% confidence intervals (CIs). The significance of the overall nonlinear association was assessed using the likelihood ratio test. A two-sided p-value < 0.05 was considered statistically significant. All statistical analyses were performed using the “rcssci” package in R software (version 4.2.1).

### 2.7 Statistical analysis

Continuous variables were expressed as mean ± standard deviation (SD), and categorical variables were represented as counts (proportions). To account for the complex sampling design of the survey, weighted statistical tests were applied based on variable type. Weighted t-tests were used to evaluate differences in continuous variables, while weighted chi-square tests were employed for categorical variables. The relationship between each individual variable and the dependent variable was examined through univariate regression analysis. Following this, a multivariable model was developed via multivariate logistic regression analysis. Odds ratios (OR) along with their 95% confidence intervals (CI) were calculated. The OR, which illustrates exposure differences between the case and control groups, was derived as (Exposed cases/Unexposed cases)/(Exposed controls/Unexposed controls). A p - value below 0.05 indicated statistical significance. All statistical analyses were carried out via R software (version 4.4.2).

## 3 Results

### 3.1 Characteristics of study participants

This study ultimately encompassed 16,948 participants, 3,319 with CKD and 13,629 without. The CKD group had a mean age of 59.02 ± 17.05, with 56.4% women, 66.4% having hypertension, an average dietary copper intake of 1.17 ± 0.71 mg, and HEI-2020 score was 51.61 ± 12.02 (**Table 1**). The non-CKD group averaged 44.45 ± 16.61 years old, with 50.6% women, 32.1% with hypertension, a mean copper intake of 1.26 ± 0.80 mg, and HEI-2020 score was 51.26 ± 12.17 (**Table 1**). **Table 1** also shows significant differences in gender, age, race, education, marital status, PIR, BMI, HPB, alcohol use, smoking status, hyperlipemia, copper intake, DKD, eGFR, uric acid levels, and HEI-2020 score between the two groups.

**Table 1:** All characteristics of the CKD and non-CKD patients.

Variable	Demographic characteristics	Overall	Non_CKD	CKD	P value
N		16948	13629	3319	
Gender (%)					0.003
	Female	8745(51.6)	6896(50.6)	1872(56.4)	
	Male	8203(48.4)	6733(49.4)	1447(43.6)	
Age (%)					
		46.86 ± 17.54	44.45 ± 16.61	59.02 ± 17.05	<0.001
	18-60	12474(73.6)	10794(79.2)	1517(45.7)	
	>=60	4474(26.4)	2835(20.8)	1802(54.3)	
Race (%)					<0.001
	Mexican American	1576(9.3)	1322(9.7)	239(7.2)	
	Non-Hispanic Black	1864(11)	1445(10.6)	431(13)	
	Non-Hispanic White	10999(64.9)	8750(64.2)	2260(68.1)	
	Other Hispanic	1034(6.1)	872(6.4)	146(4.4)	
	Other Race - Including Multi-Racial	1491(8.8)	1240(9.1)	242(7.3)	
Education (%)					<0.001
	Less than high school	712(4.2)	518(3.8)	212(6.4)	
	High school or equivalent	5288(31.2)	4143(30.4)	1162(35)	
	College or above	10948(64.6)	8968(65.8)	1945(58.6)	
Marital status (%)					<0.001
	Never married	3169(18.7)	2780(20.4)	345(10.4)	
	Married	10711(63.2)	8695(63.8)	2011(60.6)	
	Separated	3051(18)	2153(15.8)	966(29.1)	
Poverty income ratio (%)					0.002
	<1.5	4542(26.8)	3584(26.3)	972(29.3)	
	1.5-3.5	5203(30.7)	4116(30.2)	1119(33.7)	
	>3.5	7186(42.4)	5929(43.5)	1231(37.1)	
Body mass index (%)					<0.001
	<25	5051(29.8)	4239(31.1)	763(23)	
	25-30	5322(31.4)	4293(31.5)	1022(30.8)	
	>=30	6576(38.8)	5097(37.4)	1533(46.2)	
Hypertension (%)					<0.001
	No	10542(62.2)	9254(67.9)	1115(33.6)	
	Yes	6406(37.8)	4375(32.1)	2204(66.4)	
Alcohol consumption (%)					<0.001
	Never	10711(63.2)	8409(61.7)	2360(71.1)	
	Moderate	2881(17)	2385(17.5)	481(14.5)	
	Heavy	3339(19.7)	2835(20.8)	475(14.3)	
Smoking status (%)					<0.001
	Never	9847(58.1)	8055(59.1)	1766(53.2)	
	Ever	4101(24.2)	3094(22.7)	1062(32)	
	Now	3000(17.7)	2480(18.2)	491(14.8)	
Hyperlipemia (%)					<0.001
	No	3271(19.3)	2944(21.6)	345(10.4)	
	Yes	13677(80.7)	10685(78.4)	2974(89.6)	
Copper Intake					<0.001
		1.24 ± 0.78	1.26 ± 0.80	1.17 ± 0.71	
	Q1	3712(21.9)	2862(21)	866(26.1)	
	Q2	4152(24.5)	3257(23.9)	903(27.2)	
	Q3	4457(26.3)	3666(26.9)	787(23.7)	
	Q4	4627(27.3)	3843(28.2)	760(22.9)	
Diabetic kidney disease (%)					<0.001
	No	14626(86.3)	13629(100)	581(17.5)	
	Yes	898(5.3)	0(0)	1065(32.1)	
Biochemical					
	Estimated glomerular filtration rate	88.98 ± 25.35	92.68 ± 22.69	70.36 ± 29.51	<0.001
	Uric acid (umol/L)	32.95 ± 242.21	7.89 ± 5.39	159.18 ± 578.84	<0.001
HEI-2020		51.32 ± 12.14	51.26 ± 12.17	51.61 ± 12.02	0.355

Subsequently, we presented baseline characteristics of patients categorized based on their copper intake levels (**Table 2**). The analysis revealed notable disparities across these groups in terms of gender, age, ethnicity, educational background, marital status, PIR, BMI, alcohol use, smoking habits, CKD, and DKD (**Table 2**).

**Table 2:** All characteristics of the participants (grouped by copper intake).

Variable	Demographic characteristics	Overall	Q1	Q2	Q3	Q4	P value
			<0.8	0.8-1.07	1.07-1.43	>1.43	
N		16948	4238	4242	4231	4237	
Gender (%)							<0.001
	Female	8745(51.6)	2818(66.5)	2435(57.4)	2073(49)	1563(36.9)	
	Male	8203(48.4)	1420(33.5)	1807(42.6)	2158(51)	2674(63.1)	
Age (%)							0.003
		46.86 ± 17.54	45.59 ± 18.68	47.68 ± 17.83	47.08 ± 17.38	46.93 ± 16.40	
	18-60	12474(73.6)	3179(75)	3016(71.1)	3097(73.2)	3186(75.2)	
	>=60	4474(26.4)	1060(25)	1226(28.9)	1134(26.8)	1051(24.8)	
Race (%)							<0.001
	Mexican American	1576(9.3)	356(8.4)	458(10.8)	368(8.7)	390(9.2)	
	Non-Hispanic Black	1864(11)	763(18)	462(10.9)	406(9.6)	284(6.7)	
	Non-Hispanic White	10999(64.9)	2513(59.3)	2736(64.5)	2860(67.6)	2835(66.9)	
	Other Hispanic	1034(6.1)	288(6.8)	246(5.8)	250(5.9)	250(5.9)	
	Other Race - Including Multi-Racial	1491(8.8)	314(7.4)	339(8)	347(8.2)	479(11.3)	
Education (%)							<0.001
	Less than high school	712(4.2)	259(6.1)	178(4.2)	157(3.7)	136(3.2)	
	High school or equivalent	5288(31.2)	1894(44.7)	1451(34.2)	1147(27.1)	932(22)	
	College or above	10948(64.6)	2085(49.2)	2613(61.6)	2928(69.2)	3169(74.8)	
Marital status (%)							<0.001
	Never married	3169(18.7)	937(22.1)	738(17.4)	732(17.3)	784(18.5)	
	Married	10711(63.2)	2310(54.5)	2672(63)	2801(66.2)	2852(67.3)	
	Separated	3051(18)	992(23.4)	827(19.5)	698(16.5)	597(14.1)	
Poverty income ratio (%)							<0.001
	<1.5	4542(26.8)	1687(39.8)	1120(26.4)	1007(23.8)	847(20)	
	1.5-3.5	5203(30.7)	1365(32.2)	1374(32.4)	1316(31.1)	1178(27.8)	
	>3.5	7186(42.4)	1187(28)	1743(41.1)	1912(45.2)	2212(52.2)	
Body mass index (%)							<0.001
	<25	5051(29.8)	1136(26.8)	1239(29.2)	1261(29.8)	1381(32.6)	
	25-30	5322(31.4)	1267(29.9)	1260(29.7)	1358(32.1)	1419(33.5)	
	>=30	6576(38.8)	1839(43.4)	1743(41.1)	1612(38.1)	1436(33.9)	
Hyperlipemia (%)							0.074
	No	10542(62.2)	2572(60.7)	2600(61.3)	2606(61.6)	2746(64.8)	
	Yes	6406(37.8)	1666(39.3)	1642(38.7)	1625(38.4)	1491(35.2)	
Alcohol consumption (%)							0.002
	Never	10711(63.2)	2420(57.1)	2711(63.9)	2759(65.2)	2758(65.1)	
	Moderate	2881(17)	856(20.2)	670(15.8)	690(16.3)	699(16.5)	
	Heavy	3339(19.7)	962(22.7)	861(20.3)	779(18.4)	780(18.4)	
Smoking status (%)							<0.001
	Never	9847(58.1)	2289(54)	2460(58)	2526(59.7)	2542(60)	
	Ever	4101(24.2)	822(19.4)	1052(24.8)	1054(24.9)	1140(26.9)	
	Now	3000(17.7)	1127(26.6)	730(17.2)	652(15.4)	555(13.1)	
Hyperlipemia (%)							0.128
	No	3271(19.3)	720(17)	776(18.3)	859(20.3)	886(20.9)	
	Yes	13677(80.7)	3518(83)	3466(81.7)	3372(79.7)	3351(79.1)	
Chronic kidney disease (%)							<0.001
	No	14135(83.4)	3399(80.2)	3461(81.6)	3601(85.1)	3648(86.1)	
	Yes	2813(16.6)	839(19.8)	781(18.4)	630(14.9)	589(13.9)	
Diabetic kidney disease (%)							<0.001
	No	14626(86.3)	3534(83.4)	3606(85)	3702(87.5)	3758(88.7)	
	Yes	898(5.3)	301(7.1)	263(6.2)	195(4.6)	161(3.8)	
Copper intake		1.24 ± 0.78	0.62 ± 0.14	0.94 ± 0.08	1.24 ± 0.10	2.02 ± 1.11	<0.001
HEI-2020		51.32 ± 12.14	44.48 ± 10.20	49.14 ± 10.50	52.18 ± 11.43	57.91 ± 12.09	<0.001

### 3.2 Association of copper intake with CKD and DKD

We applied multivariate logistic regression to build a model evaluating copper intake’s effect on CKD, with stepwise covariate adjustments (Model 1: no covariates; Model 2: adjusted for demographics like age, gender, race, marital status, education, and PIR; Model 3: further adjusted for BMI, alcohol use, smoking, hyperlipidemia, and HBP; Model 4: further adjusted for HEI-2020 score).

In the model, when copper intake was regarded as a continuous variable, Model 1 (OR = 0.80, p < 0.001), Model 2 (OR = 0.89, p < 0.001), Model 3 (OR = 0.84, p < 0.001), and Model 4 (OR = 0.86, p = 0.003) all showed a significant negative correlation between copper intake and CKD (**Table 3**). When copper intake was considered as a categorical variable in the model, in Model 1 (Q4: OR = 0.62, p < 0.001), Model 2 (Q4: OR = 0.70, p < 0.001), Model 3 (Q4: OR = 0.58, p < 0.001), and Model 4 (Q4: OR = 0.60, p < 0.001), it was found that the Q4 group had a significantly lower risk of developing CKD compared to the Q1 group (**Table 3**).

**Table 3:** Association of copper intake with CKD.

Copper intake	Model 1	P value	Model 2	P value	Model 3	P value	Model 4	P value
	OR (95%CI)		OR (95%CI)		OR (95%CI)		OR (95%CI)	
Q1								
Q2	0.90 (0.81-1.00)	0.047	0.88 (0.78-0.99)	0.028	0.84 (0.71-1.01)	0.058	0.84 (0.71-1.01)	0.061
Q3	0.75 (0.67-0.83)	<.001	0.80 (0.71-0.90)	<.001	0.73 (0.61-0.87)	<.001	0.73 (0.61-0.88)	<.001
Q4	0.62 (0.56-0.70)	<.001	0.70 (0.61-0.79)	<.001	0.58 (0.48-0.71)	<.001	0.60 (0.49-0.73)	<.001
p for trend	0.80 (0.75-0.85)	<.001	0.89 (0.83-0.95)	<.001	0.84 (0.76-0.93)	<.001	0.86 (0.77-0.95)	0.003

For DKD, similar results were observed. As a continuous variable, copper intake was significantly negatively associated with DKD in all models: Model 1 OR = 0.73 (p <0.001), Model 2 OR = 0.81 (p < 0.001), Model 3 OR = 0.84 (p = 0.018), and Model 4 OR = 0.87 (p = 0.016). As a categorical variable, Q4 individuals had a significantly lower risk of DKD compared to Q1 in all models: Model 1 (Q4: OR = 0.52, p < 0.001), Model 2 (Q4: OR = 0.57, p < 0.001), Model 3 (Q4: OR = 0.58, p < 0.001), and Model 4 (Q4: OR = 0.60, p < 0.001) (**Table 4**).

**Table 4:** Association of copper intake with DKD.

Copper intake	Model 1	P value	Model 2	P value	Model 3	P value	Model 4	P value
	OR (95%CI)		OR (95%CI)		OR (95%CI)		OR (95%CI)	
Q1								
Q2	0.82 (0.71-0.96)	0.01	0.81 (0.69-0.96)	0.016	0.83 (0.66-1.05)	0.13	0.86 (0.71-1.05)	0.15
Q3	0.64 (0.55-0.75)	<.001	0.68 (0.56-0.81)	<.001	0.63 (0.50-0.81)	<.001	0.72 (0.58-0.88)	0.002
Q4	0.52 (0.44-0.61)	<.001	0.57 (0.47-0.69)	<.001	0.58 (0.44-0.75)	<.001	0.60 (0.48-0.75)	<.001
p for trend	0.73 (0.65-0.82)	<.001	0.81 (0.72-0.91)	<.001	0.84 (0.73-0.97)	0.018	0.87 (0.77-0.97)	0.016

### 3.3 Subgroup analysis of the association between copper intake and CKD and DKD

We performed subgroup analyses based on age, gender, BMI, alcohol consumption, smoking status, diabetes mellitus, HBP, hyperlipidemia, and DKD. In all subgroups, the Q4 group showed a lower risk of CKD than the Q1 group in Model 1 (**Table 5**). Specific results were as follows: male (Q4: OR = 0.57, p < 0.001), female (Q4: OR = 0.67, p < 0.001), age 18-60 (Q4: OR = 0.80, p = 0.006), age ≥ 60 (Q4: OR = 0.53, p < 0.001), non-DKD (Q4: OR = 0.69, p = 0.003), BMI < 25 (Q4: OR = 0.67, p < 0.001), BMI ≥ 30 (Q4: OR = 0.77, p = 0.002), BMI 25-30 (Q4: OR = 0.51, p < 0.001), never drinking alcohol (Q4: OR = 0.57, p < 0.001), moderate drinking alcohol (Q4: OR = 0.66, p = 0.029), never smoking (Q4: OR = 0.65, p < 0.001), ever smoking (Q4: OR = 0.41, p < 0.001), non-diabetes (Q4: OR = 0.60, p < 0.001), diabetes (Q4: OR = 0.58, p < 0.001), HBP (Q4: OR = 0.57, p < 0.001), non-hyperlipidemia (Q4: OR = 0.49, p < 0.001), and hyperlipidemia (Q4: OR = 0.53, p < 0.001).

**Table 5:** Subgroup analysis of the association between copper intake and CKD.

Subgroup	Stratum	Copper intake	Model 1	P value	Model 2	P value	Model 3	P value
			OR (95%CI)		OR (95%CI)		OR (95%CI)	
Gender	Male							
		Q1						
		Q2	0.92 (0.79-1.08)	0.33	1.02 (0.84-1.23)	0.859	0.90 (0.69-1.17)	0.436
		Q3	0.76 (0.65-0.89)	<.001	0.91 (0.75-1.10)	0.312	0.74 (0.57-0.96)	0.024
		Q4	0.57 (0.49-0.67)	<.001	0.73 (0.61-0.89)	0.002	0.56 (0.43-0.74)	<.001
	Female							
		Q1						
		Q2	0.87 (0.76-0.99)	0.04	0.80 (0.69-0.94)	0.005	0.77 (0.61-0.98)	0.036
		Q3	0.71 (0.61-0.82)	<.001	0.72 (0.61-0.85)	<.001	0.70 (0.54-0.90)	0.006
		Q4	0.67 (0.57-0.78)	<.001	0.70 (0.58-0.84)	<.001	0.59 (0.44-0.79)	<.001
Age	18-60							
		Q1						
		Q2	0.87 (0.74-1.02)	0.089	0.84 (0.70-1.01)	0.061	0.76 (0.60-0.97)	0.025
		Q3	0.79 (0.67-0.93)	0.005	0.86 (0.72-1.03)	0.101	0.75 (0.59-0.95)	0.016
		Q4	0.80 (0.68-0.94)	0.006	0.83 (0.69-1.00)	0.046	0.66 (0.51-0.85)	0.001
	>=60							
		Q1						
		Q2	0.86 (0.74-1.00)	0.043	0.88 (0.75-1.03)	0.121	0.84 (0.67-1.05)	0.132
		Q3	0.69 (0.59-0.81)	<.001	0.73 (0.61-0.86)	<.001	0.67 (0.53-0.85)	<.001
		Q4	0.53 (0.45-0.62)	<.001	0.57 (0.47-0.68)	<.001	0.52 (0.40-0.66)	<.001
DKD	No							
		Q1						
		Q2	1.07 (0.85-1.34)	0.577	0.98 (0.77-1.26)	0.895	0.92 (0.68-1.25)	0.6
		Q3	0.86 (0.68-1.08)	0.196	0.87 (0.67-1.12)	0.282	0.80 (0.59-1.09)	0.159
		Q4	0.69 (0.53-0.88)	0.003	0.71 (0.53-0.93)	0.014	0.60 (0.43-0.85)	0.004
	Yes							
		Q1						
		Q2	1.00 (0.00-Inf)	1	0.98 (0.77-1.26)	0.895	0.92 (0.68-1.25)	0.6
		Q3	1.00 (0.00-Inf)	1	0.87 (0.67-1.12)	0.282	0.80 (0.59-1.09)	0.159
		Q4	1.00 (0.00-Inf)	1	0.71 (0.53-0.93)	0.014	0.60 (0.43-0.85)	0.004
BMI	<25							
		Q1						
		Q2	1.00 (0.80-1.23)	0.974	1.05 (0.82-1.36)	0.688	0.87 (0.57-1.34)	0.537
		Q3	0.73 (0.59-0.92)	0.007	0.89 (0.69-1.16)	0.399	0.73 (0.48-1.11)	0.142
		Q4	0.67 (0.54-0.84)	<.001	0.80 (0.62-1.05)	0.11	0.55 (0.35-0.86)	0.009
	>=30							
		Q1						
		Q2	1.02 (0.88-1.19)	0.778	0.94 (0.79-1.11)	0.455	0.95 (0.74-1.22)	0.685
		Q3	0.86 (0.73-1.01)	0.062	0.85 (0.71-1.01)	0.072	0.78 (0.61-1.01)	0.064
		Q4	0.77 (0.65-0.91)	0.002	0.76 (0.63-0.93)	0.008	0.62 (0.47-0.82)	<.001
	25-30							
		Q1						
		Q2	0.72 (0.60-0.87)	<.001	0.69 (0.55-0.86)	<.001	0.66 (0.48-0.91)	0.011
		Q3	0.67 (0.55-0.81)	<.001	0.68 (0.55-0.85)	<.001	0.61 (0.44-0.84)	0.002
		Q4	0.51 (0.42-0.62)	<.001	0.56 (0.45-0.71)	<.001	0.49 (0.35-0.69)	<.001
Alcohol use	Never							
		Q1						
		Q2	0.95 (0.82-1.11)	0.524	0.89 (0.75-1.06)	0.19	0.90 (0.71-1.14)	0.37
		Q3	0.73 (0.63-0.85)	<.001	0.73 (0.61-0.87)	<.001	0.62 (0.49-0.80)	<.001
		Q4	0.57 (0.49-0.67)	<.001	0.60 (0.50-0.72)	<.001	0.53 (0.41-0.68)	<.001
	Moderate							
		Q1						
		Q2	0.76 (0.54-1.09)	0.139	0.79 (0.53-1.18)	0.245	0.66 (0.37-1.20)	0.176
		Q3	0.90 (0.64-1.27)	0.549	1.01 (0.69-1.48)	0.969	1.03 (0.59-1.82)	0.909
		Q4	0.66 (0.46-0.96)	0.029	0.74 (0.49-1.12)	0.155	0.78 (0.42-1.43)	0.421
	Heavy							
		Q1						
		Q2	0.82 (0.59-1.14)	0.241	1.03 (0.70-1.51)	0.9	0.73 (0.42-1.27)	0.262
		Q3	0.89 (0.64-1.23)	0.482	1.18 (0.81-1.73)	0.386	0.88 (0.51-1.53)	0.654
		Q4	0.78 (0.56-1.08)	0.135	1.13 (0.76-1.69)	0.532	1.00 (0.57-1.77)	0.996
Smoking status	Never							
		Q1						
		Q2	0.89 (0.77-1.02)	0.093	0.83 (0.71-0.98)	0.03	0.73 (0.54-0.98)	0.039
		Q3	0.69 (0.60-0.80)	<.001	0.70 (0.59-0.84)	<.001	0.62 (0.45-0.84)	0.002
		Q4	0.65 (0.56-0.75)	<.001	0.70 (0.58-0.83)	<.001	0.64 (0.46-0.88)	0.006
	Ever							
		Q1						
		Q2	0.75 (0.61-0.91)	0.004	0.78 (0.62-0.97)	0.026	0.96 (0.68-1.37)	0.822
		Q3	0.62 (0.51-0.76)	<.001	0.72 (0.58-0.91)	0.005	0.61 (0.43-0.87)	0.007
		Q4	0.41 (0.33-0.50)	<.001	0.52 (0.41-0.66)	<.001	0.48 (0.33-0.70)	<.001
	Now							
		Q1						
		Q2	0.95 (0.74-1.21)	0.669	1.06 (0.80-1.39)	0.697	1.05 (0.66-1.65)	0.841
		Q3	0.95 (0.73-1.22)	0.674	1.19 (0.89-1.60)	0.228	1.25 (0.79-1.99)	0.34
		Q4	0.79 (0.60-1.03)	0.081	0.90 (0.66-1.23)	0.51	0.87 (0.52-1.44)	0.582
Diabetes	No							
		Q1						
		Q2	1.01 (0.79-1.28)	0.951	0.99 (0.76-1.30)	0.951	1.00 (0.74-1.36)	0.992
		Q3	0.74 (0.58-0.95)	0.019	0.79 (0.60-1.05)	0.099	0.76 (0.56-1.04)	0.089
		Q4	0.60 (0.46-0.78)	<.001	0.67 (0.50-0.91)	0.009	0.67 (0.48-0.93)	0.018
	Yes							
		Q1						
		Q2	0.78 (0.65-0.95)	0.012	0.79 (0.64-0.97)	0.026	0.79 (0.60-1.03)	0.08
		Q3	0.64 (0.52-0.78)	<.001	0.67 (0.54-0.84)	<.001	0.66 (0.50-0.87)	0.003
		Q4	0.58 (0.47-0.71)	<.001	0.59 (0.46-0.75)	<.001	0.60 (0.45-0.80)	<.001
Blood pressure	No							
		Q1						
		Q2	0.90 (0.74-1.08)	0.248	0.82 (0.66-1.01)	0.058	0.66 (0.47-0.94)	0.021
		Q3	0.80 (0.66-0.96)	0.019	0.79 (0.64-0.98)	0.03	0.71 (0.51-0.99)	0.043
		Q4	0.86 (0.71-1.03)	0.102	0.86 (0.70-1.07)	0.182	0.62 (0.43-0.88)	0.008
	Yes							
		Q1						
		Q2	0.94 (0.82-1.07)	0.36	0.92 (0.79-1.06)	0.245	0.91 (0.74-1.11)	0.345
		Q3	0.77 (0.67-0.88)	<.001	0.81 (0.70-0.95)	0.009	0.71 (0.57-0.88)	0.002
		Q4	0.57 (0.50-0.66)	<.001	0.61 (0.52-0.72)	<.001	0.54 (0.43-0.68)	<.001
Hyperlipemia	No							
		Q1						
		Q2	0.85 (0.59-1.22)	0.366	0.82 (0.54-1.23)	0.333	0.88 (0.53-1.46)	0.615
		Q3	0.73 (0.50-1.04)	0.083	0.80 (0.53-1.22)	0.309	0.89 (0.53-1.50)	0.658
		Q4	0.49 (0.33-0.73)	<.001	0.56 (0.35-0.88)	0.012	0.65 (0.38-1.11)	0.117
	Yes							
		Q1						
		Q2	0.85 (0.74-0.97)	0.017	0.81 (0.70-0.95)	0.008	0.84 (0.69-1.01)	0.066
		Q3	0.67 (0.58-0.77)	<.001	0.72 (0.62-0.84)	<.001	0.69 (0.57-0.84)	<.001
		Q4	0.53 (0.45-0.61)	<.001	0.58 (0.49-0.69)	<.001	0.56 (0.45-0.69)	<.001

### 3.4 Potential nonlinear relationship between copper intake and CKD and DKD

**Figure 2:**
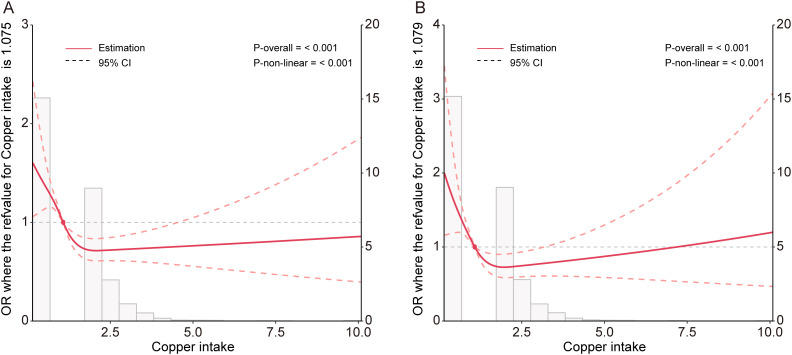
Potential nonlinear relationship between copper intake and CKD **(A)**, and DKD **(B)** in the Model 3.

The restricted cubic spline plots derived from the fully adjusted model (Model 3) were utilized to analyze the nonlinear relationship between copper intake and CKD) as well as DKD. The analysis produced a p-value for nonlinearity of less than 0.001, indicating a U-shaped relationship (**Figures 2A, B**). This finding suggests a significant nonlinear correlation between copper intake and both CKD and DKD.

We constructed a piecewise linear regression model, using 1.47 mg as the inflection point to categorize participants into two groups: those with copper intake ≤ 1.47 mg and those with copper intake > 1.47 mg. We found that copper intake was negatively correlated with CKD in the group with copper intake ≤ 1.47 mg (**Table 6**, OR: 0.51, p < 0.001). Additionally, we constructed another piecewise linear regression model, using 0.98 mg as the inflection point to further categorize participants into two groups: those with copper intake ≤ 0.98 mg and those with copper intake > 0.98 mg. Copper intake was also inversely correlated with DKD among participants with a copper intake ≤ 0.98 mg (**Table 7**, OR = 0.40, p = 0.021).

**Table 6:** Association between copper intake and CKD based on piecewise linear regression analysis in model 3.

Copper intake	Adjust OR (95%CI)	Pvalue
≤1.47	0.51 (0.39-0.66)	<.001
>1.47	1.00 (0.90-1.10)	0.997

**Table 7:** Association between copper intake and DKD based on piecewise linear regression analysis in model 3.

Copper intake	Adjust OR (95%CI)	Pvalue
<=0.98	0.40 (0.19-0.87)	0.021
>0.98	1.00 (0.88-1.12)	0.956

## 4 Discussion

This study was studied cross-sectionally under DRC and DKD populations in the NHANES database. The purpose of this study was to investigate the relationship between copper records and the occurrence of CKD and DKD. The results of this study indicated an inverse association between copper intake and CKD and DKD, which was consistent among different subgroups.

CKD is a globally prevalent health issue characterized by a gradual and irreversible decline in kidney function, typically persisting for more than three months. CKD can be attributed to various diseases or conditions, with diabetes and hypertension being the most common contributors (1). Prolonged elevated blood sugar levels can damage the small blood vessels in the kidneys, leading to impaired filtration capabilities. About 30% of people with -type 1 diabetes and 40% of people with -type 2 diabetes have DKD (24, 25). DKD is a significant cause of CKD and end-stage renal disease (ESRD). It not only affects renal function by disrupting the normal processes of waste and excess fluid removal from the body but is also associated with increased mortality rates (26, 27). While various treatment options are available for CKD and DKD (28, 29), current therapies are only able to delay the progression of these diseases and their efficacy is restricted. Research indicates that patients with CKD frequently exhibit imbalances in essential trace elements (30). These imbalances are not only common complications of CKD but also significant risk factors for disease progression, cardiovascular events, and mortality (30). Accordingly, understanding the relationship between dietary intake of essential trace elements and the risk of CKD and DKD may provide valuable insights. These insights could contribute to improved management and slowing of disease progression in affected patients.

Copper, a vital micronutrient, is critical for physiological processes like antioxidant defense and energy metabolism (31–33). However, Too much copper consumption can lead to its buildup in the kidneys, resulting in nephrotoxicity. This is characterized by oxidative stress, cell damage, and necrosis of the proximal tubules, ultimately resulting in a decline in renal function (16). The relationship between copper and kidney disease is bidirectional. CKD patients are prone to copper homeostasis imbalance due to impaired renal excretion and altered protein metabolism (34). Ahmad et al. have found that high blood copper levels are linked to a higher prevalence of CKD and kidney function decline (18). As a cofactor for superoxide dismutase, copper deficiency in CKD patients may reduce enzyme activity, thereby increasing the risk of oxidative damage (30). Meanwhile, excessive copper can lead to mitochondrial damage and tissue inflammation. Copper deficiency can also accelerate atherosclerosis by affecting lipid metabolism, further impacting renal function (30). Recent studies have found higher urinary copper concentrations in DKD patients compared to non-DKD individuals (19). In DKD, maintaining copper balance is critical. Copper deficiency can reduce the activity of antioxidant enzymes (35). Conversely, excess copper may exacerbate kidney damage by inducing oxidative stress and inflammatory responses (35). Both scenarios can ultimately impair renal function. Our study found that the average dietary copper intake was 1.17 mg in CKD populations and 1.26 mg in non-CKD populations. Moreover, a significant inverse association was observed between copper intake and CKD at levels ≤ 1.47 mg, and with DKD at intake levels ≤ 0.98 mg. This indicates that keeping copper levels within the appropriate range may play a role in maintaining kidney health and mitigating the risk of CKD and DKD. It is important to note that copper intake is closely linked to broader dietary patterns; for instance, copper-rich foods such as nuts, seeds, and whole grains are often components of a generally healthy diet (10). Therefore, we incorporated the HEI-2020 as a covariate in a multivariate model (Model 4) to account for overall diet quality. The significant inverse association between dietary copper intake and the prevalence of CKD and DKD persisted even after further adjustment for overall diet quality using the HEI-2020 in Model 4. This robustness enhances the credibility of our findings by suggesting that the protective association is not merely a surrogate for a generally healthy diet. Instead, it points to a potential independent role of adequate copper intake, within the context of a balanced diet, in reducing the risk of kidney disease.

This study leveraged large-scale data from the NHANES, with key strengths including the use of a nationally representative sample, appropriate weighting methods accounting for the complex survey design, rigorous adjustment for potential confounders through multiple models, and the application of RCS to uncover a U-shaped nonlinear relationship between copper intake and both CKD and DKD. Notably, a protective association was observed within specific intake thresholds (≤1.47 mg for CKD and ≤0.98 mg for DKD), and this association remained consistent across various subgroups. However, several limitations should be acknowledged. First, the cross-sectional design precludes the inference of causality between copper intake and CKD/DKD, and reverse causality cannot be ruled out. Second, dietary copper intake was assessed using 24-hour recall methods, which are subject to recall bias and day-to-day variability, thus may not accurately reflect long-term habitual intake. Although multiple potential confounders were adjusted for in the analyses, residual confounding due to unmeasured or unknown factors (e.g., other dietary components) may still exist. Furthermore, as the study sample was derived from a U.S. population, caution is warranted when generalizing the findings to other ethnic, geographic, or clinical populations.

A significant inverse association was observed between copper intake and the risk of both CKD and DKD within specific thresholds (≤1.47 mg for CKD and ≤0.98 mg for DKD).

## Data Availability

The original contributions presented in the study are included in the article/**Supplementary Material**. Further inquiries can be directed to the corresponding author.

## Supplementary material

Supplementary Table 1 Scoring criteria for the Healthy Eating Index-2020 (HEI-2020).
